# Air Pollution Exposure—A Trigger for Myocardial Infarction?

**DOI:** 10.3390/ijerph7041486

**Published:** 2010-03-31

**Authors:** Niklas Berglind, Petter Ljungman, Jette Möller, Johan Hallqvist, Fredrik Nyberg, Mårten Rosenqvist, Göran Pershagen, Tom Bellander

**Affiliations:** 1 Institute of Environmental Medicine, Karolinska Institutet, SE-171 77 Stockholm, Sweden; E-Mails: petter.ljungman@ki.se (P.L.); fredrik.nyberg@ki.se (F.N.); goran.pershagen@ki.se (G.P.); tom.bellander@ki.se (T.B.); 2 Bristol-Myers Squibb Research and Development, Pennington, NJ 08534, USA; 3 Department of Cardiology, Karolinska Institutet, Stockholm South Hospital, SE-118 83 Stockholm, Sweden; E-Mail: marten.rosenqvist@sodersjukhuset.se; 4 Department of Public Health Sciences, Karolinska Institutet, SE-171 77 Stockholm, Sweden; E-Mails: jette.moller@ki.se (J. M.); johan.hallqvist@pubcare.uu.se (J.H.); 5 Department of Public Health and Caring Sciences, Uppsala University, SE-751 22 Uppsala, Sweden; 6 AstraZeneca R&D, SE-431 83 Mölndal, Sweden

**Keywords:** air pollution, myocardial infarction, trigger, onset, case cross-over design

## Abstract

The association between ambient air pollution exposure and hospitalization for cardiovascular events has been reported in several studies with conflicting results. A case-crossover design was used to investigate the effects of air pollution in 660 first-time myocardial infarction cases in Stockholm in 1993–1994, interviewed shortly after diagnosis using a standard protocol. Air pollution data came from central urban background monitors. No associations were observed between the risk for onset of myocardial infarction and two-hour or 24-hour air pollution exposure. No evidence of susceptible subgroups was found. This study provides no support that moderately elevated air pollution levels trigger first-time myocardial infarction.

## 1. Introduction

Ambient air pollution has been associated with an increase in hospitalization for cardiovascular and respiratory events in several studies [1–3]. Most large studies have relied on rather broad outcome definitions, such as cardiorespiratory morbidity [4], cardiovascular morbidity [1,3], or cardiac morbidity [5–8]. A few studies have investigated more specific endpoints, including ventricular arrhythmias [9–11], congestive heart failure [12], and myocardial infarction [13–20]. By studying specific diagnoses it is possible to gain more knowledge of the mechanisms behind how air pollution exposure is related to morbidity.

Although many studies have investigated the association between air pollution and myocardial infarction, only three studies [13,16,17] have used sufficiently precise information on the time of onset of myocardial infarction to be able to study the immediate temporal relationship between air pollution exposure and onset of myocardial infarction using a case-crossover design. In 2001, Peters *et al.* showed a clear positive relationship between preceding 2-hour increases in PM2.5 and the onset of myocardial infarction in Boston, MA, while in 2005, Peters *et al.* from Augsburg, Germany, and Sullivan *et al.* from Seattle, WA, showed little or no effect of air pollution exposure on the onset of myocardial infarction. In the Boston study, detailed interviews with each subject immediately followed their myocardial infarction, while the Augsburg and Seattle studies relied on information collected for myocardial infarction registers. The interviews performed in the Boston study enabled a very accurate determination of the time of onset of the myocardial infarction, including information about premonitory symptoms. Misclassification of MI onset time would be expected to bias the health effects estimates towards the null [21]. Therefore, it remains unclear whether the positive results from the Boston study are due to the uniquely high quality data available on time of MI onset in that study, or to other differences between studies.

The present study made use of detailed interviews of persons suffering from their first myocardial infarction in Stockholm, Sweden, providing well-characterized data on risk factors and hypothesized triggers of myocardial infarction and the onset of symptoms. The methodology was similar to that used in the study from Boston. We specifically aimed to explore the influence of a short duration of air pollution exposure immediately preceding myocardial infarction and possible modification of this effect by other risk factors.

## 2. Materials and Methods

### 2.1. Study Population

The Swedish Onset study is described in detail elsewhere [22–25]. In brief, the Onset study is a case-crossover study nested in a large population based case-control study of causes of myocardial infarction—the Stockholm Heart Epidemiology Program (SHEEP). Myocardial infarction cases, defined as persons with first events of myocardial infarction, were recruited from April 1993 to December 1994 from all 10 emergency hospitals in the region using a designated organization and from continuous screening of death certificates by Statistics Sweden. Most cases were directly identified in the coronary care units of the departments of internal medicine at nine of the 10 regional hospitals. A small number of missed cases were identified later through the computerized hospital discharge registry and were then included. All cases of hospitalized myocardial infarction were diagnosed according to standardized criteria using information on symptoms, electrocardiograms, and enzyme levels. The study base included all Swedish citizens aged 45–70 years who had not had a previous myocardial infarction and were living in Stockholm County. Cases were included at the time of disease incidence.

Out of the 1,489 cases in the study area during the study period, 495 deceased before recruitment or were too sick to participate, 210 were not identified or recruited, 21 were cared for in hospitals out of the study area and 64 did not want to participate. That left us with 699 patients interviewed for the case-crossover study. An important consideration was to determine the exact timing of onset; therefore this information was collected both from the medical records and from the interview. After exclusion of patients with unreliable information on time of onset or with a high percentage of missing or clearly inaccurate answers, 660 cases remained for analysis. The cases were not specifically chosen based on survivor status, but since inclusion was conditional on the ability to be interviewed, the likelihood of including fatal myocardial infarctions was small. As it turned out, all included cases were alive 28 days after onset of the myocardial infarction; hence we refer to them as non-fatal myocardial infarctions.

### 2.2. Interview

The cases were interviewed during their hospital stay or shortly afterwards. The timing of the interview ranged from the day of the myocardial infarction to more than four months after, with a median interval of 15 days between myocardial infarction onset and the interview. The interviews were carried out by 28 nurses who had received preparatory training during at least three evening classes. Meetings for discussion of methodological problems were held regularly during the study period. The median duration of the interview was 90 minutes.

At the interview, detailed information was first obtained on all episodes of pain (clock time, type, duration, *etc*.), other symptoms, and circumstances during the four days before the myocardial infarction to determine the precise time of disease onset. The interviewers were also instructed to distinguish between premonitory symptoms of disease onset and symptoms of ordinary angina pectoris.

Comprehensive information on common cardiovascular disease risk factors was available from the postal questionnaire of the SHEEP Study that was delivered after the Onset interview. The response rate of the questionnaire was 91% among cases already interviewed in the Onset study. Disease history, the presence of other risk factors for cardiovascular disease, and regular medication use were determined from the questionnaire information and from measurements made at the SHEEP health examination (generally performed three months after diagnosis when a metabolically stable status was assumed).

### 2.3. Air Pollution and Meteorology

Air pollutants available for the study period were PM_10_, NO_2_, CO, and O_3_. Air pollution and meteorological data were obtained from fixed centrally located roof-top monitors reflecting urban background levels for PM_10_, NO_2_, and CO, and from rurally located monitors for O_3_ representing regional background levels. Monitoring of PM10 started in March 1994 and was available from a single monitor. NO_2_, CO, and O_3_ values were available from multiple monitoring sites and a city mean was calculated. Prior to calculation of the city mean, missing hourly values for each monitor were imputed using the method described by Berglind *et al.* [26]. In constructing the 24-hour averages, a minimum of 75% of air pollution data was required for inclusion in the statistical analysis. Both time points were required for calculating two-hour averages. In the construction of averages, complete air pollution data were required for the two-hour averages, and a maximum of 25% of missing hourly air pollution data was allowed for 24-hour averages.

### 2.4. Statistical Analysis

The association between the onset of myocardial infarction and air pollution exposure was analyzed using a case-crossover design [27]. In this design, the time period immediately preceding the time of the event is considered the case period and other time periods when no events occurred are considered control periods for that subject. In this manner, each case serves as its own control and covariates that are constant within a subject during the studied time period are adjusted for by design. The association is then analyzed using a conditional logistic regression model. The end of the case period was defined as the onset time of myocardial infarction rounded down to the nearest preceding hour. Time-stratified control period selection was performed by matching control periods to the case period on time of day, day of week, calendar month, and year within each subject, resulting in three or four control periods for each case period [28]. Air pollution levels and meteorological data were averaged in two-hour and 24-hour windows preceding the time of onset for the case period and the corresponding time for the control periods. Each conditional logistic regression model included a linear term for air pollution and penalized splines for temperature and relative humidity for the same averaging time as the air pollution parameter. In exploration of potential effect modification, an interaction term between the air pollution parameter and the potential effect modifier was included in the regression model. All associations are expressed as odds ratios with 95% confidence intervals for an interquartile range (IQR) increase in mean concentration for each pollutant and averaging time. No adjustment for multiplicity was performed. All data management and summary statistics were performed using Stata Version 9 and all conditional regression modeling was performed using S-plus version 7.

## 2. Results

The vast majority of the cases were males and 75% were current or previous smokers (Table 1). The distribution of the air pollution parameters are presented in Table 2 for one- and 24-hour time periods. Monitoring of PM_10_ did not begin until March 1994 and was thus only available for approximately half of the study period. The most highly correlated pollutants were CO and NO_2_ (r = 0.74 for 24-hour means).

Table 3 shows the estimated odds ratios for the risk of onset of myocardial infarction for an increase of one IQR in each of the air pollution measures. The associations for all pollutants and both averaging periods were all close to unity.

**Figure 1.**
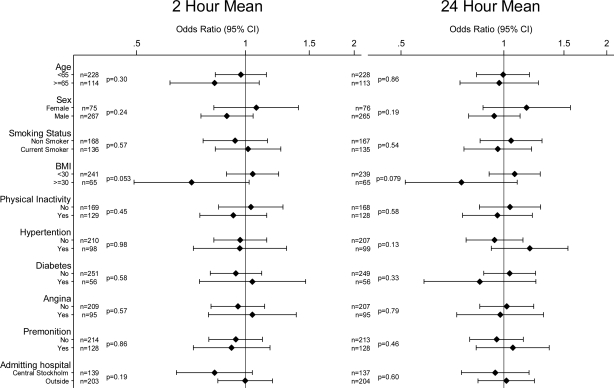
Odds ratios of myocardial infarction for an interquartile range increase in 2 h and 24 h moving averages of PM_10_ in different subgroups (P-values for interaction). Number of observations for each subgroup with complete data on analyzed covariate and air pollution indicated by n.

**Figure 2.**
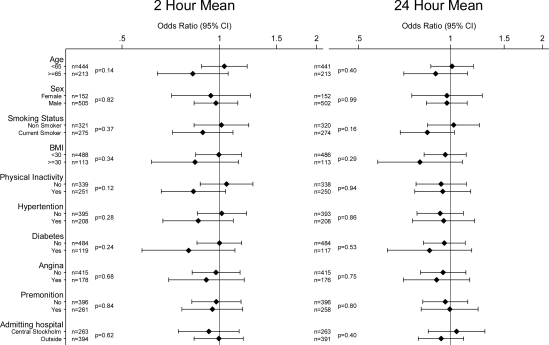
Odds ratios of myocardial infarction for an interquartile range increase in 2 h and 24 h moving averages of NO_2_ in different subgroups (P-values for interaction). Number of observations for each subgroup with complete data on analyzed covariate and air pollution indicated by n.

**Figure 3.**
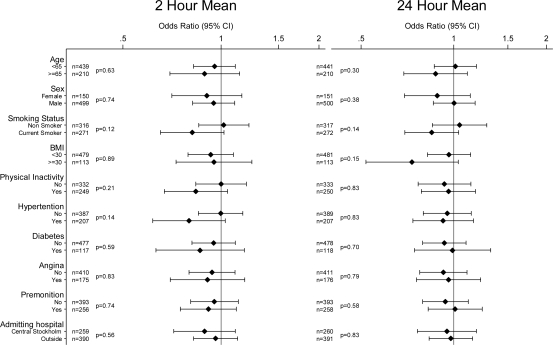
Odds ratios of myocardial infarction for an interquartile range increase in 2 h and 24 h moving averages of CO in different subgroups (P-values for interaction). Number of observations for each subgroup with complete data on analyzed covariate and air pollution indicated by n.

**Figure 4.**
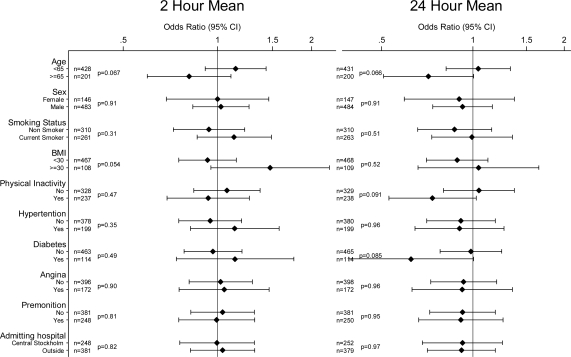
Odds ratios of myocardial infarction for an interquartile range increase in 2 h and 24 h moving averages of O_3_ in different subgroups (P-values for interaction). Number of observations for each subgroup with complete data on analyzed covariate and air pollution indicated by n.

There was no clear evidence of effect modification with any of the investigated variables for any of the pollutants or averaging times. Potential effect modifiers investigated were: age; sex; smoking status; BMI; physical inactivity; a history of hypertension, diabetes, and angina; the presence or absence of premonition; and the location of the admitting hospital (Central Stockholm or elsewhere) Figures 1 to 4 show odds ratios and 95% confidence intervals for the onset of myocardial infarction for an IQR increase in each of the air pollutants for two- and 24-hour measures by potential effect modifiers.

## 4. Discussion

Our study did not show associations between two-hour or 24-hour air pollution levels and the onset of myocardial infarction. This is in conflict with the first study from Boston [13] but in line with the findings from Augsburg [16] and Seattle [17]. Analyses of possible effect modifiers used in previous studies, as well as of others not previously examined, did not show any associations for any of the air pollutants in any subgroup and none of the interactions between air pollutants and the covariates was statistically significant at the 0.05 level. This is remarkable in light of the fact that we estimated a total of 168 odds ratios and performed 80 interaction tests without any correction for multiplicity. The pollutants are correlated with each other and so are some of the modifiers, reducing the number of truly independent estimates. Even so, it would not have been surprising had we found a few estimates where the 95% confidence interval excluded unity.

As mentioned earlier, there are three previously reported studies that used similar temporal precision as we did when investigating the relationship between air pollution exposure and the onset of myocardial infarction. In order to compare our study in more detail with the other three, we provide a comparison of background characteristics in Table 4 and air pollution levels and main results in Table 5. The adjusted odds ratios presented in Table 5 are scaled to show the OR for an increase of the same unit increase across all four studies. There are several differences between the four studies. In comparing the effect estimates the Boston study stands out when it comes to the particle exposure even though showing levels similar to other studies. For the other pollutants all four studies report similar effect estimates. In contrast to the other investigated cities, Boston is in an area in which fine PM is dominated by sulfate. The sulfates are formed from sulfur dioxide emitted mainly from coal-burning power plants and other industrial sources, such as smelters, industrial boilers, and oil refineries. Although sulfates are regarded as relatively harmless in themselves, they may function as an indicator of a harmful mixture of air pollutants. The largest difference between the present study and the previously reported studies was that we chose to focus solely on persons experiencing their first myocardial infarction. Roughly a third of patients in Boston and Seattle had previously experienced a myocardial infarction and 14% of patients in Augsburg. We also had a larger proportion of current smokers and lower proportions of patients who had previously experienced angina pectoris or had been diagnosed with diabetes or hypertension. Peters *et al.* argued that the larger proportion of hypertensive patients in Augsburg might diminish the association between air pollution and myocardial infarction; however no mechanism for this was suggested. Our study population was slightly healthier than those of the other studies in terms of previous heart disease and diabetes, and one could speculate that this makes them less sensitive to air pollution as a trigger for myocardial infarction. A distinction in susceptibility for air pollution with respect to severity of myocardial infarction has been seen for long term and short-term exposure to air pollution where exposure seems to be more strongly associated to fatal myocardial infarction, especially occurring out of hospital [19,29–31]. All cases in our study were non-fatal.

This study offered the opportunity of a well-defined study population with detailed information characterizing the onset of myocardial infarction collected within a few days after diagnosis. Air pollution data was collected continuously and retrieved retrospectively, blinded to the outcome. Some limitations need to be acknowledged. The main limitation is the lack of particulate air pollution data. Only PM_10_ was available and only for less than half of the study period. It would have been desirable to have data for finer particles like PM_2.5_ and particle number concentrations in order to better compare our results with the other studies and also to address the concern that these particles are particularly important for the cardiovascular effects of air pollution. In a previous analysis from Stockholm regarding the onset of ventricular arrhythmias in response to two-hour increases in air pollution [11], the distance between where the event occurred and the air pollution monitor influenced the observed association. This raises the concern over the influence of misclassification of exposure when using fixed monitors in collecting air pollution exposure data. In an effort to examine this we performed interaction analyses based on the location of the admitting hospital, under the assumption that a subject who was admitted to a hospital in central Stockholm was in the city when the MI occurred, and hence the exposure information from the centrally located monitors may be more representative of the actual exposure compared to a subject who was admitted to a hospital farther away from the monitors. This interaction analysis did not provide any evidence that the potential misclassification of exposure due to distance from the air pollution monitors is responsible for the lack of a positive association. The Stockholm Onset study has previously been used to analyze several other triggers for myocardial infarction and associations have been observed for episodes of anger, heavy physical exertion, sexual activity, and work related stressful life events [22–25]. It is not likely that any one of these factors would act as a confounder for the association between exposure to air pollution and acute onset of myocardial infarction. In addition, although this study was comparable in size to both the Boston and Augsburg studies, it was small in comparison to the Seattle study and the possibility of false negative results remains a concern.

In summarizing the evidence from all four studies, there appears to be weak support of a rapid effect of air pollution in triggering the onset of myocardial infarction. However, evidence from other studies showing specific ischemic associations must be considered in the overall interpretation. A meta-analysis of 19 time-series studies [32] showed a 0.8% increased risk (95% CI 0.6, 1.1) of hospital admissions for ischemic heart disease with a 10 μg/m^3^ short-term increase of PM_10_. Animal models have demonstrated effects of air pollution on inflammation [33], oxidative stress [34] and endothelial dysfunction [35] including plaque progression and destabilization in an animal model of atherosclerosis [36]. In double-blind randomized crossover studies performed on humans in exposure chamber, ST-segment depression [37] as well as thrombus formation and platelet activation [38] have been demonstrated after 1–2-hour exposures to diesel exhaust. ST-segment depressions have also been shown in a panel study in the presence of ambient levels of PM_2.5_ and black soot [39]. Short-term increases in air pollution were associated with unstable angina or acute myocardial infarction in patients undergoing cardiac catherization with stronger associations seen in multi-vessel coronary disease [40]. With the exception of the meta-analysis of time-series studies, all of the above mentioned human studies investigated patients with established coronary artery disease, suggesting that this may be an important factor modifying the ischemic response to air pollution. Therefore, total absence of a real association between air pollution and myocardial infarction seems unlikely, although it may be restricted to some extent to subgroups. Future efforts might explore air pollution as a trigger for myocardial infarction in patients with established coronary disease and perhaps focus on fatal events occurring out of hospital which seem to be particularly susceptible to air pollution increases.

## 5. Conclusions

We did not see any associations between short-term increases in air pollution levels and the onset of myocardial infarction in Stockholm. This study does not support a short-term elevation in the risk of first-time myocardial infarction onset at moderately elevated air pollution levels.

## Figures and Tables

**Table 1.** Characteristics of myocardial infarction cases in the Stockholm Onset study (n = 660).

Age in years		Current Smoker	276 / 598 (46%)
Mean (SD)	60 (7.2)	Previous Smoker	174 / 598 (29%)
Median (range)	61 (44, 70)	Physical inactivity	252 / 592 (43%)
Age >= 65 years	214 / 660 (32%)	Hypertension	209 / 605 (35%)
Male	507 / 660 (77%)	Diabetes	120 / 605 (20%)
BMI >= 30	113 / 603 (19%)	Angina	178 / 595 (30%)

Table shows n / N (%) except for age in years.

**Table 2.** Hourly and daily distribution of air pollution and meteorology parameters in Stockholm, April 1993 to December 1994.

							Correlation			
Parameter (unit)	n	mean	SD	median	25%	75%	PM_10_	NO_2_	CO	O_3_
**1 hour**										
PM_10_ (ug/m^3^)*	7,107*	18.2	12.3	15.3	10.0	22.7	1.0			
NO_2_ (ug/m^3^)	15,323	26.0	14.5	23.4	14.9	34.0	0.29	1.0		
CO (mg/m^3^)	15,198	0.51	0.30	0.44	0.31	0.63	0.29	0.72	1.0	
O_3_ (ug/m^3^)	14,618	56.8	22.8	56.4	41.1	71.8	0.31	−0.18	−0.16	1.0
Temp (°C)	15,343	8.0	7.8	8.0	2.1	13.7	0.09	−0.17	−0.16	0.36
Rel. hum. (%)	15,343	70.4	17.9	74.0	57.8	85.2	−0.17	0.06	0.18	−0.61
**24-hours**										
PM_10_ (ug/m^3^)*	299*	18.1	9.4	15.5	11.9	21.5	1.0			
NO_2_ (ug/m^3^)	640	25.9	9.5	24.9	19.2	31.5	0.23	1.0		
CO (mg/m^3^)	637	0.51	0.19	0.47	0.39	0.60	0.26	0.74	1.0	
O_3_ (ug/m^3^)	617	56.9	17.3	56.5	44.9	68.4	0.50	−0.19	−0.32	1.0
Temp (°C)	640	8.0	7.4	8.3	2.2	13.6	0.10	−0.26	−0.32	0.33
Rel. hum. (%)	640	70.4	14.1	71.9	59.1	82.2	−0.25	0.09	0.37	−0.63

* Monitoring of PM_10_ began in March 1994.

**Table 3.** Association of air pollutants with onset of myocardial infarction. Estimated odds ratios (OR) are adjusted for temperature and relative humidity.

Pollutant	IQR	No. of cases	OR	95% CI
**2-hour average**				
PM_10_ (ug/m^3^)*	12.2	342	0.93	(0.81, 1.08)
NO_2_ (ug/m^3^)	18.1	657	0.97	(0.84, 1.11)
CO (mg/m^3^)	0.32	649	0.94	(0.82, 1.07)
O_3_ (ug/m^3^)	29.6	629	1.02	(0.85, 1.23)
**24-hour average**				
PM_10_ (ug/m^3^)*	9.1	341	0.99	(0.85, 1.15)
NO_2_ (ug/m^3^)	12.3	654	0.97	(0.85, 1.11)
CO (mg/m^3^)	0.23	651	0.97	(0.85, 1.11)
O_3_ (ug/m^3^)	24.7	631	0.92	(0.75, 1.13)

* Monitoring for PM_10_ began in March 1994.

**Table 4.** Characteristics of myocardial infarction cases: a comparison of four studies.

Study center	Study period	N	Mean age	Median age	Male	Prior MI	Prior angina	Diabetes	Hypertension	Current smoker
Boston	1995–1996	772	62	-	63%	31%	23%	19%	41%	32%
Seattle	1988–1994	5793	-	69	67%	31%	41%	18%	49%	23%
Augsburg	1999–2000	691	60	-	77%	14%	24%	21%	66%	36%
Stockholm	1993–1994	660	60	61	68%	0%	19%	13%	36%	39%

**Table 5.** Air pollution profiles and effect estimates for air pollution on onset of myocardial infarction. A comparison of four studies.

Pollutant	City	1-hour mean	1-hour IQR	1-hour 5–95%	Averaging time	Adjusted OR (95% CI)†
PM_2.5_	Boston	12.1		27.0	2-hour	1.17 (1.04, 1.32)
	Seattle	12.8	10.6		2-hour	1.01 (0.97, 1.05)
	Augsburg	16.3	9.1		1-hour	0.98 (0.87, 1.11)
PM_10_	Boston	19.4		39.2	2-hour	1.11 (1.01, 1.21)
	Seattle	28.3	20.5			
	Augsburg				Same day*	1.02 (0.97, 1.06)
	Stockholm	18.2	12.7	37.5	2-hour	0.94 (0.84, 1.07)
CO	Boston	1.3		2.1	2-hour	1.02 (0.99, 1.05)
	Seattle	2.3	1.6		2-hour	1.00 (1.00, 1.01)
	Augsburg	0.5	0.4		Same day	1.00 (0.97, 1.03)
	Stockholm	0.5	0.3	0.9	2-hour	0.98 (0.95, 1.02)
NO_2_	Boston	43.2		75.2	2-hour	1.01 (0.96, 1.06)
	Augsburg	35.8	20.0		Same day	1.03 (0.97, 1.10)
	Stockholm	24.4	19.1	46.9	2-hour	0.98 (0.91, 1.06)
O_3_	Boston	38.8		88.2	2-hour	1.03 (0.98, 1.08)
	Augsburg	43.5	48.5		Same day	1.00 (0.95, 1.05)
	Stockholm	56.1	30.7	74.8	2-hour	1.01 (0.95, 1.07)

* From February 1999 to December 1999, PM10 was estimated from total suspended particles (TSP) by multiplying the TSP measurement by a factor of 0.83.

† Boston adjusted for season, meteorology and day of week, Seattle and Stockholm adjusted for relative humidity and temperature, Augsburg did not specify for case-crossover analyses. All effect estimates are expressed per 10 μg/m^3^ increment except for CO which are expressed per 0.1mg/m^3^ increment.
